# Endoplasmic Reticulum Stress in Hypertension and Salt Sensitivity of Blood Pressure

**DOI:** 10.1007/s11906-024-01300-9

**Published:** 2024-04-11

**Authors:** Maria Balhara, Kit Neikirk, Andrea Marshall, Antentor Hinton, Annet Kirabo

**Affiliations:** 1 Department of Medicine, Division of Clinical Pharmacology, Vanderbilt University Medical Center, Nashville, TN 37212-8802 USA; 2 Department of Molecular Physiology and Biophysics, Vanderbilt University, Nashville, TN 37232 USA; 3 Vanderbilt Center for Immunobiology, Nashville, USA; 4 Vanderbilt Institute for Infection, Immunology and Inflammation, Nashville, USA; 5 Vanderbilt Institute for Global Health, Nashville, USA

**Keywords:** Hypertension, ER stress, UPR, Autophagy

## Abstract

**Purpose of Review:**

Hypertension is a principal risk factor for cardiovascular morbidity and mortality, with its severity exacerbated by high sodium intake, particularly in individuals with salt-sensitive blood pressure. However, the mechanisms underlying hypertension and salt sensitivity are only partly understood. Herein, we review potential interactions in hypertension pathophysiology involving the immune system, endoplasmic reticulum (ER) stress, the unfolded protein response (UPR), and proteostasis pathways; identify knowledge gaps; and discuss future directions.

**Recent Findings:**

Recent advancements by our research group and others reveal interactions within and between adaptive and innate immune responses in hypertension pathophysiology. The salt-immune-hypertension axis is further supported by the discovery of the role of dendritic cells in hypertension, marked by isolevuglandin (IsoLG) formation. Alongside these broadened understandings of immune-mediated salt sensitivity, the contributions of T cells to hypertension have been recently challenged by groups whose findings did not support increased resistance of Rag-1-deficient mice to Ang II infusion. Hypertension has also been linked to ER stress and the UPR. Notably, a holistic approach is needed because the UPR engages in crosstalk with autophagy, the ubiquitin proteasome, and other proteostasis pathways, that may all involve hypertension.

**Summary:**

There is a critical need for studies to establish cause and effect relationships between ER stress and the UPR in hypertension pathophysiology in humans and to determine whether the immune system and ER stress function mainly to exacerbate or initiate hypertension and target organ injury. This review of recent studies proposes new avenues for future research for targeted therapeutic interventions.

**Supplementary Information:**

The online version contains supplementary material available at 10.1007/s11906-024-01300-9.

## Introduction

Hypertension is implicated in approximately 10 million deaths (95% UI, 9.6–11.8 million) annually worldwide; it also contributes significantly to the burden of heart disease, stroke, and kidney failure [1]. The salt sensitivity of blood pressure is characterized by a pronounced blood pressure elevation after sodium intake. It significantly affects cardiovascular morbidity and mortality in substantial proportions of hypertensive and normotensive adults [2, 3•]. Despite extensive research, the pathophysiology of hypertension, particularly salt-sensitive hypertension, is not fully understood.

Beyond the view of hypertension as a consequence of salt retention, vascular abnormalities, and neurogenic dysfunction, research has increasingly focused on the role of the immune system. In this context, findings have revealed that both adaptive and innate immunity are important contributors to pathophysiological changes. Immune dysregulation and inflammation are associated with hypertensive pathology. For example, Guzik et al. identified a previously undefined role of T lymphocytes as critical mediators of angiotensin II (AngII)-induced hypertension and vascular dysfunction [4–7]. However, contrasting recent findings, such as those from Senuik et al. and Bode et al., which also included RAG1-deficient mice, as used in Guzik et al.’s original studies, indicate that a more complete understanding of these relationships is needed [8–12, 13•, 14•, 15•]. Research has also shown the contributions of CD8+ T cells, CD4+ T cells, regulatory T cells, and B cells to hypertension [16–19]. Shah et al. reported that myeloid-derived suppressor cell depletion promotes T-cell activity and the onset and progression of hypertension [20]. In addition to adaptive immunity, studies of innate immunity performed by De Ciuceis et al. and others have revealed how macrophages, monocytes, and the NLRP3 inflammasome contribute to hypertension [21–23].

We and our collaborators have built on these findings to further examine the salt-immune-hypertension axis. Kirabo et al. identified a previously undefined role of monocyte-derived dendritic cells (DCs) in hypertension by finding that under hypertensive conditions, DCs initiate a cascade of events during which the elevated oxidative environment promotes the formation and accumulation of γ-ketoaldehydes or isolevuglandins (IsoLGs) [24]. These IsoLGs rapidly create adducts with lysine residues on proteins. DCs then present these adducts as neoantigens that T cells recognize as nonself, triggering an immune response and contributing to the progression of hypertension. Subsequent research in our laboratory further delineated the sodium-induced immune activation pathway, demonstrating that Na^+^ enters DCs via the amiloride-sensitive epithelial sodium channel (ENaC) during a process controlled by serum/glucocorticoid regulated kinase 1 (SGK1). The resulting calcium influx and activation of NADPH oxidase produces IsoLGs that participate in T-cell activation [25, 26]. We also found that this ENaC-mediated IsoLG production in DCs promotes the formation and activation of the NLRP3 inflammasome. These findings increase the understanding of a critical innate immune pathway that contributes to hypertension [27•].

The role of oxidative stress in immune activation highlights the need to examine other forms of cellular stress (e.g., misfolded protein accumulation in the endoplasmic reticulum (ER)) for potential interconnected effects on immune activation and hypertension progression. The role of the ER in the development or exacerbation of hypertension is a growing area of interest in cardiovascular pathophysiology. During physiological stress, disruption of ER function due to the accumulation of misfolded or unfolded proteins can trigger a cascade of maladaptive responses. However, there are gaps in the literature regarding ER-mediated pathologies, including whether ER disruption is a cause or a consequence of hypertension, specific mechanisms, crosstalk with other cellular stress response pathways, and potential new drug targets. This review examines these knowledge gaps, summarizes ER pathway studies in hypertension, and identifies directions for future research.

## Endoplasmic Reticulum Stress and the Unfolded Protein Response

**Fig. 1:**
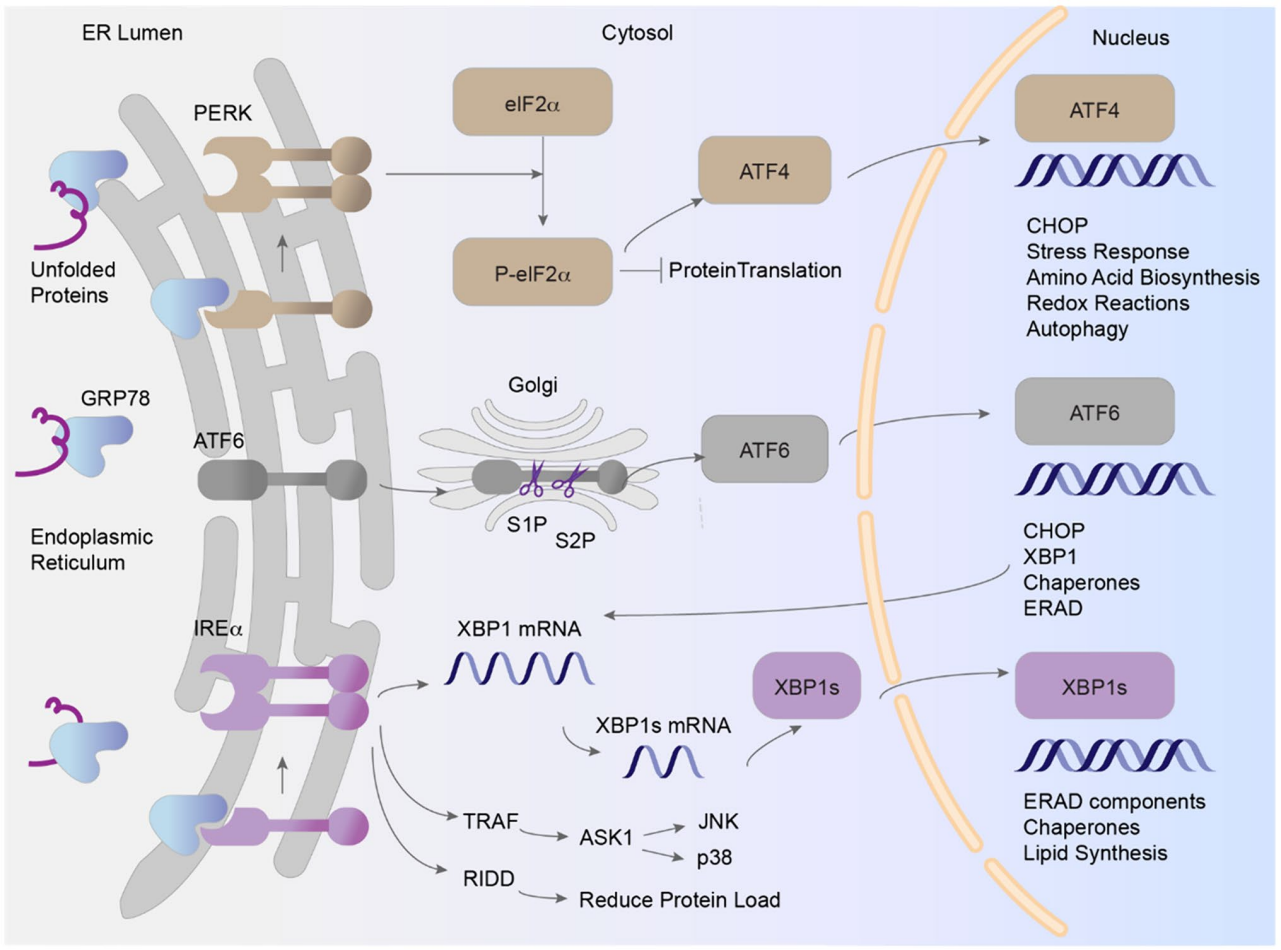
The PERK, IRE1α, and ATF6 pathways are central to the unfolded protein response (UPR), which mitigates ER stress caused by misfolded proteins. ASK1, Apoptosis Signal-Regulating Kinase 1; ATF4, Activating Transcription Factor 4; ATF6, Activating Transcription Factor 6; CHOP, C/EBP Homologous Protein; eIF2α, Eukaryotic Initiation Factor 2 Alpha; ERAD, Endoplasmic Reticulum-Associated Degradation; GRP78, Glucose-Regulated Protein 78; IRE1α, Inositol-Requiring Enzyme 1 Alpha; JNK, c-Jun N-terminal Kinase; p38, p38 Mitogen-Activated Protein Kinases; P-eIF2α, Phosphorylated Eukaryotic Initiation Factor 2 Alpha; PERK, PKR-Like Endoplasmic Reticulum Kinase; RIDD, Regulated IRE1-Dependent Decay; S1P, Site-1 Protease; S2P, Site-2 Protease; TRAF, TNF Receptor-Associated Factor; XBP1, X-Box Binding Protein 1; XBP1s, Spliced X-Box Binding Protein 1

The ER is a cellular organelle involved in protein synthesis and folding, lipid biosynthesis, and intracellular calcium regulation. Physiological and external stressors can disrupt ER function and lead to ER stress, which is the accumulation of unfolded or misfolded proteins in the ER (Fig. 1) [28]. In separate studies, Mori et al. and Cox et al. described the unfolded protein response (UPR), a previously unknown process that cells use to resolve ER stress [29, 30]. The UPR mitigates ER stress by halting protein translation, increasing chaperone expression, and activating degradation pathways.

The UPR can be protective or detrimental, depending on the cellular context and the intensity and duration of ER stress. Under acute, resolvable ER stress, the UPR restores ER homeostasis and promotes cell survival. However, under severe, unremitting ER stress, the adaptive capacity of the UPR becomes overwhelmed, and the UPR initiates apoptosis [31–33]. This delicate balance between adaptive and apoptotic responses indicates the critical role of the UPR in cellular physiology and disease pathogenesis.

Studies have revealed the mechanisms of the UPR in health and disease. Initially, studies revealed that the levels of glucose-regulated proteins (GRPs), such as GRP78, increase during glucose deprivation and in response to misfolded proteins in the ER [34–38]. Mori et al. and Cox et al. identified inositol-requiring enzyme 1 alpha (IRE1α), a kinase protein on the ER membrane that functions as a sensor for ER stress and upregulates the transcription of GRP78 and other chaperones in response to misfolded protein accumulation [29, 30]. Haze et al. subsequently identified activating transcription factor 6 (ATF6) [39], and Harding et al. and Shi et al. identified protein kinase R (PKR)-like ER kinase (PERK) [40, 41]; both of these proteins function as ER stress sensors. Under nonstressed conditions, GRP78 binds to and inactivates PERK, IRE1α, and ATF6. When the ER is stressed due to increased levels of misfolded proteins, GRP78 dissociates from these sensor proteins and binds to misfolded proteins, acting as a chaperone. This dissociation activates PERK, IRE1α, and ATF6, which initiates the UPR [42].

Shi et al. and Harding et al. reported that upon activation, PERK phosphorylates eukaryotic initiation factor 2 alpha (eIF2α); this change leads to a translational block that reduces overall protein synthesis and thus alleviates the protein folding burden in the ER [40, 41, 43]. Other studies have shown that PERK selectively increases the translation of activating transcription factor 4 (ATF4), which induces the expression of the proapoptotic transcription factor C/EBP homologous protein (CHOP; also known as GADD153) and continues to be translated from upstream open reading frames, even when phosphorylated eIF2α inhibits general protein synthesis [44–46].

**Fig. 2:**
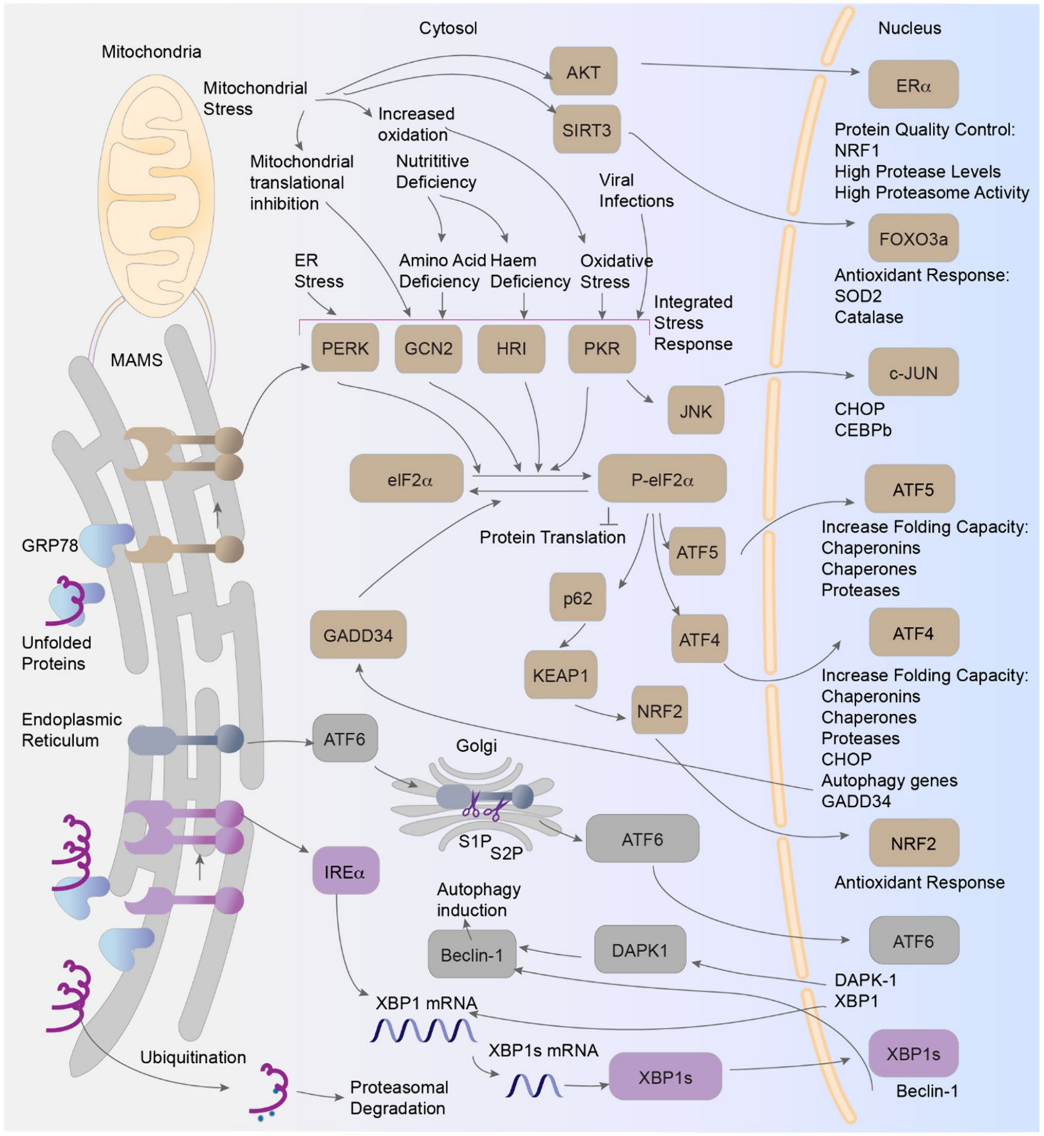
Crosstalk between the UPR and other proteostasis pathways. AKT, Protein Kinase B; ATF4, Activating Transcription Factor 4; ATF5, Activating Transcription Factor 5; ATF6, Activating Transcription Factor 6; CEBPb, CCAAT/Enhancer Binding Protein Beta; CHOP, C/EBP Homologous Protein; DAPK-1, Death-Associated Protein Kinase 1; eIF2α, Eukaryotic Initiation Factor 2 Alpha; ERα, Estrogen Receptor Alpha; FOXO3a, Forkhead Box O3; GADD34, Growth Arrest and DNA Damage-Inducible Protein 34; GCN2, General Control Nonderepressible 2; GRP78, Glucose-Regulated Protein 78; HRI, Heme-Regulated Inhibitor; IRE1α, Inositol-Requiring Enzyme 1 Alpha; JNK, c-Jun N-terminal Kinase; KEAP1, Kelch-Like ECH-Associated Protein 1; MAMS, Mitochondria-Associated Membranes; NRF1, Nuclear Respiratory Factor 1; P-eIF2α, Phosphorylated Eukaryotic Initiation Factor 2 Alpha; PERK, PKR-Like Endoplasmic Reticulum Kinase; PKR, Protein Kinase R; S1P, Site-1 Protease; S2P, Site-2 Protease; SIRT3, Sirtuin 3; SOD2, Superoxide Dismutase 2; TRAF, TNF Receptor-Associated Factor; XBP1, X-Box Binding Protein 1; XBP1s, Spliced X-Box Binding Protein 1

Yoshida et al. reported that upon activation, IRE1α cleaves X-Box binding protein 1 (XBP1) mRNA to generate spliced XBP1 mRNA (XBP1s) [47]. XBP1s is a highly active transcription factor that translocates to the nucleus and drives gene expression to increase the protein folding capacity of the ER, promote ER membrane expansion, and drive ER-associated degradation (ERAD). Holien et al. revealed that IRE1α also modulates a separately regulated Ire1-dependent decay (RIDD) pathway that degrades mRNAs encoding ER-targeted proteins, thus reducing folding demand [48]. Urano et al. and others reported that IRE1α can initiate apoptotic pathways by binding to tumor necrosis factor receptor-associated factor 2, activating signal-regulating kinase 1, c-Jun NH2-terminal kinase, and p38 mitogen-activated protein kinase [49]. Han et al. reported that IRE1α can initiate apoptosis through prolonged regulation of Ire1-dependent decay-mediated degradation of chaperone protein mRNAs [50], further highlighting the role of IRE1α in the UPR balance (Fig. 2).

Ye et al. reported that upon activation, ATF6 is cleaved by site-1 protease (S1P) and site-2 protease (S2P) enzymes to generate an active ATF6 fragment. This ATF6 processing is completely blocked in S2P-deficient cells and partially blocked in S1P-deficient cells [51]. As S1P is localized in the Golgi, Chen et al. subsequently demonstrated that activated ATF6 translocates to the Golgi, where it is cleaved by S1P and S2P [52]. The resulting active ATF6 fragment then translocates to the nucleus and enhances gene expression to augment the protein folding capacity of the ER and increase ERAD. The accumulation of misfolded proteins within the ER lumen is thus reduced.

## Crosstalk between the Unfolded Protein Response and Other Proteostasis Pathways

The UPR interacts with proteostasis pathways, including cytoplasmic stress responses, such as autophagy, the ubiquitin proteasome pathway (UPP), the heat shock response (HSR), and the integrated stress response (ISR); and organelle-specific stress responses, such as the mitochondrial UPR (UPRmt).

### Autophagy

The autophagy pathway degrades and recycles misfolded proteins into autophagosomes. Autophagy is positively regulated by the phosphoinositide 3-kinase complex and AMP-activated protein kinase and negatively regulated by the mammalian target of rapamycin 1. Margariti et al. and others reported that the expression of Beclin-1, a component of the phosphoinositide 3-kinase complex, is upregulated by the binding of XBP1s to its promoter [53]; CHOP and c-Jun NH2-terminal kinase-mediated reductions in the inhibitory effect of B-cell lymphoma 2 (Bcl-2) on Beclin-1 [54, 55]; and ATF6-mediated upregulation of death-associated protein kinase 1 (DAPK1), which in turn phosphorylates and activates Beclin-1 [56, 57], stimulating autophagy. Høyer-Hansen et al. and others reported that increased ER Ca2+ release during ER stress upregulates AMP-activated protein kinase, leading to autophagy [58–60]. Jin et al. revealed that ATF4 upregulates Regulated in Development and DNA Damage Responses 1, which subsequently downregulates the mammalian target of rapamycin 1, stimulating autophagy [61].

The UPR upregulates critical autophagy genes. Rzymski reported that ATF4 enhances LC3B expression [62]. B'Chir et al. showed that ATF4 and CHOP activate p62 and other autophagy genes [63]. Autophagy interacts bidirectionally with the UPR. Adolph et al. reported that dysfunction in either the UPR or autophagy leads to compensatory increases in the other [64]. ER-phagy, an ER-specific form of autophagy, is critical for delivering ER fragments to autophagosomes for degradation, thereby maintaining ER function [65]. Because hypertension has been linked to dysfunction in the ER stress response, compounds that stimulate autophagy or ER-phagy could enhance misfolded protein clearance and ER function, potentially relieving ER stress and reducing hypertension-related damage.

### Ubiquitin Proteasome Pathway

Within the UPP pathway, misfolded proteins in the ER are retrotranslocated by ERAD to the cytosol and then tagged by E3 ubiquitin ligases for 26S proteasome degradation. Because both the UPP and autophagy support proteostasis via proteolytic activity, balanced crosstalk occurs between them. Zhu et al. and Zheng et al. reported that pharmacological proteasome inhibition leads to autophagy upregulation [66, 67]. Conversely, Wang et al. reported that acute autophagy inhibition increases proteasomal subunit expression and proteasomal activity [68]. However, Korolchuk et al. reported that during chronic autophagy inhibition, UPP impairment due to p62 accumulation delays the delivery of ubiquitinated substrates to the proteasome [69]. Therefore, long-term dysfunction in both the UPP and the autophagy pathway, as occurs during aging, results in the accumulation of misfolded proteins, leading to ER stress. Preventing dysfunction in these proteolytic pathways is crucial for efficient clearance of misfolded proteins from the ER, potentially affecting ER stress and hypertension.

### Heat Shock Response

Liu et al. reported that ER stress can induce HSR, although the effect of ER stress-induced HSR is less pronounced than that of heat stress-induced HSR. Conversely, HSR activation by constitutively active heat shock factor 1 (Hsf1) partially rescues defects in ERAD and ER-to-Golgi transport in UPR-deficient ire1D cells. Thus, the HSR can assist the UPR by supporting ERAD and ER protein transport [70]. Kennedy et al. demonstrated that by downregulating the Bcl-2 interacting mediator of cell death (BIM), a proapoptotic Bcl-2 homology 3 domain-only (BH3-only) protein, the HSR can have an inhibitory effect on ER stress-induced apoptosis [71]. Therefore, the HSR has implications for ER stress-related conditions and hypertension.

### Integrated Stress Response

EIF2α phosphorylation initiates the ISR in response to various stress conditions. Phosphorylation can be mediated by different kinases, including PERK (protein misfolding), general control nonderepressible 2 (GCN2; amino acid deprivation), PKR (viral infection), and heme-regulated inhibitor (iron deficiency). Alasiri et al.’s study revealed compensatory mechanisms among these kinases [72]. Specifically, GCN2 silencing leads to a corresponding increase in PERK expression; this response suggests that PERK is upregulated to offset the absence of GCN2 in a negative feedback loop. PERK inhibition increases GCN2 expression, which indicates that GCN2 is upregulated to offset PERK loss [72]. Zhu et al. showed that the UPR can be triggered by viral infection and, once activated, leads to increased PKR expression, revealing an interaction between the UPR and the ISR [73]. ISR activity can thus cooperate with ER activity and potentially affect ER stress and hypertension.

### Mitochondrial Unfolded Protein Response

The accumulation of unfolded or misfolded proteins in the mitochondria initiates the mitochondrial UPR, which maintains mitochondrial proteostasis, ensures efficient energy production, and limits reactive oxygen species (ROS) generation. Mitochondria-associated ER membranes enable lipid and Ca2+ exchange between the ER and mitochondria. Advances in imaging may yield quantified analyses of these membranes and offer insights into their architecture and involvement in hypertension [74–77]. These analyses are critical because early UPR activity enhances ER–mitochondria coupling, increasing Ca2^+^ uptake and ATP production [78]. However, severe ER stress results in mitochondrial Ca2^+^ overload, which causes mitochondrial fragmentation and apoptosis [79]. The UPR affects mitochondrial function, primarily via PERK. Verfaille et al. reported that PERK-deficient cells have weaker ER-mitochondria contact sites [80]. Hori et al. revealed that the expression of the Lon protease, which degrades oxidatively damaged proteins in mitochondria, is enhanced by PERK [81]. Bouman et al. reported that the PERK-ATF4 pathway upregulates Parkin, a significant regulator of mitochondrial function [82]. Ichimura et al. and others found that PERK-mediated phosphorylation of p62 enhances its binding to Kelch-Like ECH-associated protein 1 (KEAP1), leading to activation of nuclear factor erythroid 2-related factor 2 (NRF2); NRF2 is the master regulator of the cellular antioxidant response [83, 84]. Mitochondria also reciprocally affect the UPR. Duplan et al. reported that Parkin represses p53 and upregulates XBP-1 [85]. By influencing ER function, the mitochondrial UPR can potentially have implications for various conditions, including hypertension.

Crosstalk among the different stress response pathways that interact with ER function is essential for maintaining homeostasis. Disruption of the balance between these pathways is associated with many disease states. Because these stress response systems mediate proteostasis, they can compensate for each other. This characteristic suggests that ER stress can be mitigated by balancing alternative pathways via targeted interventions. In this context, emerging single-cell technologies (e.g., imaging combined with metabolomics) offer significant promise [86, 87]. These technologies allow for an in-depth examination of the interorganellar trafficking of molecules. Thus, these methods provide powerful approaches for understanding the complex crosstalk between different cellular components and pathways.

## Cross-Involvement of the Unfolded Protein Response with Other Roles of the Endoplasmic Reticulum

The ER is essential for lipid biosynthesis and intracellular calcium regulation. It is a primary site for the production of sterols and phospholipids, which are significant components of biological membranes. Activation of the UPR via ER stress alters the expression of lipid metabolism genes. PERK and eIF2 phosphorylation activate sterol regulatory element-binding protein (SREBP)-1c and SREBP-2. ATF6 modulates SREBP-2, suppressing lipid-related gene transcription while increasing SREBP-1c expression. ATF4 upregulates stearoyl-CoA desaturase 1, fatty acid synthase, acetyl-CoA carboxylase, and SREBP-1c. Conversely, CHOP and IRE1 downregulate C/EBPα and C/EBPβ activity. XBP1s modulates stearoyl-CoA desaturase 1, diacylglycerol-O-acyltransferase 2, and acetyl-CoA carboxylase expression [88].

**Fig. 3:**
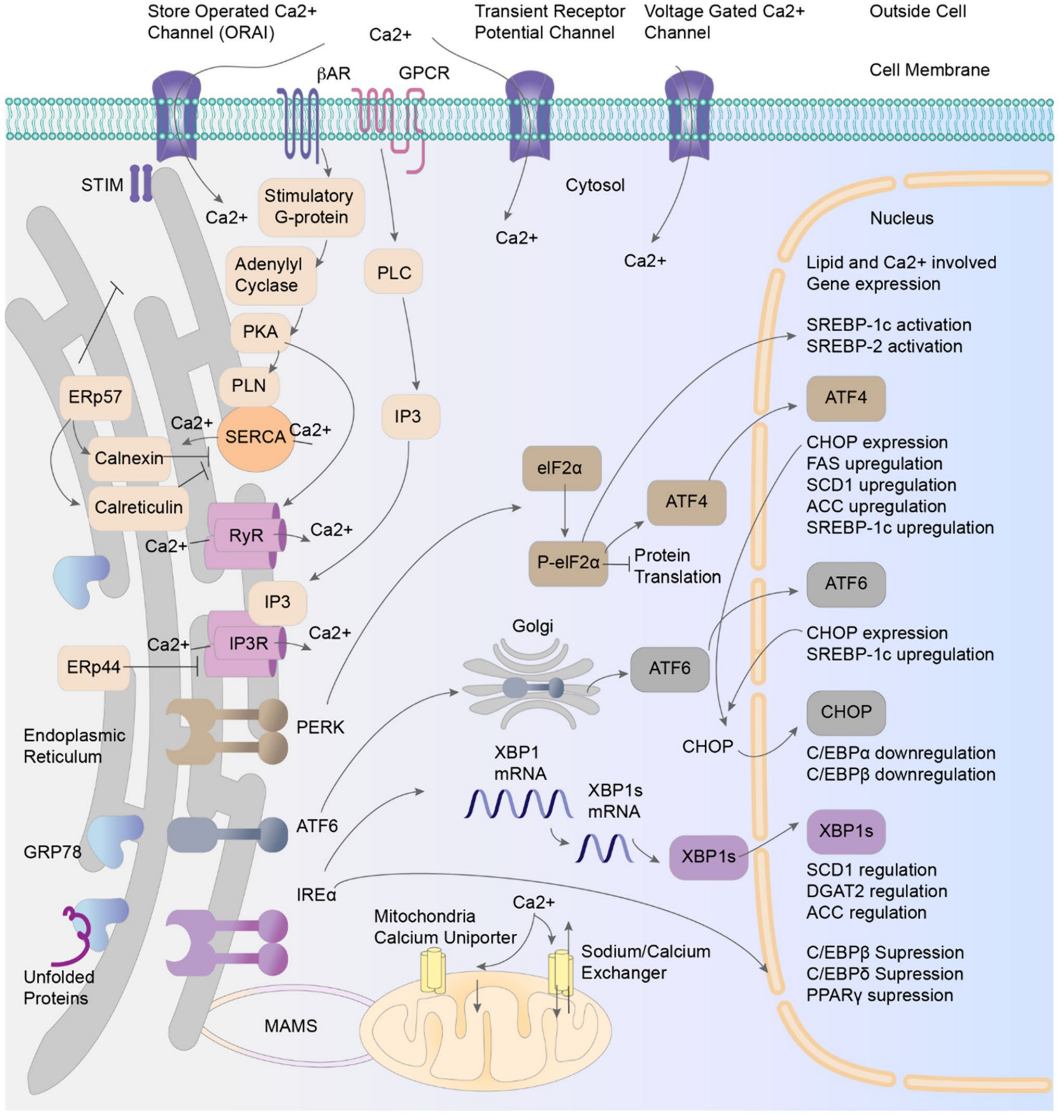
Crosstalk between ER proteins and Ca2 + and lipid homeostasis in the UPR. ACC, Acetyl-CoA Carboxylase; ATF4, Activating Transcription Factor 4; ATF6, Activating Transcription Factor 6; C/EBPα, CCAAT/Enhancer Binding Protein Alpha; C/EBPβ, CCAAT/Enhancer Binding Protein Beta; C/EBPδ, CCAAT/Enhancer Binding Protein Delta; Ca2 + , Calcium Ion; CHOP, C/EBP Homologous Protein; DGAT2, Diacylglycerol O-acyltransferase 2; eIF2α, Eukaryotic Initiation Factor 2 Alpha; ERp44, Endoplasmic Reticulum Protein 44; ERp57, Endoplasmic Reticulum Protein 57; FAS, Fatty Acid Synthase; GRP78, Glucose-Regulated Protein 78; IP3, Inositol 1,4,5-Trisphosphate; IP3R, IP3 Receptor; IRE1α, Inositol-Requiring Enzyme 1 Alpha; MAMS, Mitochondria-Associated Membranes; ORAI, Calcium Release-Activated Calcium Modulator 1; P-eIF2α, Phosphorylated Eukaryotic Initiation Factor 2 Alpha; PERK, PKR-like Endoplasmic Reticulum Kinase; PKA, Protein Kinase A; PLC, Phospholipase C; PLN, Phospholamban; PPARγ, Peroxisome Proliferator-Activated Receptor Gamma; RyR, Ryanodine Receptor; SCD1, Stearoyl-CoA Desaturase 1; SERCA, Sarco/Endoplasmic Reticulum Ca2 + -ATPase; SREBP-1c, Sterol Regulatory Element-Binding Protein 1c; SREBP-2, Sterol Regulatory Element-Binding Protein 2; STIM, Stromal Interaction Molecule; XBP1, X-Box Binding Protein 1; XBP1s, Spliced X-Box Binding Protein 1

Cytosolic calcium homeostasis requires a precise balance between the influx of extracellular calcium through voltage-gated and ligand-gated channels and the release of intracellular calcium from stores such as those in the ER (Fig. 3) [89, 90]. The release of intracellular Ca2^+^ from ER stores is thus an important component of cellular calcium homeostasis. ER Ca2^+^ concentrations are maintained by sarcoplasmic/ER Ca2^+^-ATPase, which actively transports Ca2^+^ from the cytosol to the ER, creating a calcium gradient. Within the ER, protein-folding chaperones such as GRP78, calnexin, and calreticulin serve as Ca2^+^-binding proteins that buffer Ca2^+^ concentrations. Calreticulin is a major Ca2^+^-binding protein that maintains 50% of the total ER Ca2^+^ concentration [91]. Ca2^+^ is released from the ER to the cytosol via two types of ER membrane-resident channels: inositol 1,4,5-trisphosphate receptors and ryanodine receptors. Under ER stress, Ca2+ release from the ER lumen into the cytosol can disrupt calcium homeostasis [92]. ER stress can thus affect lipid and calcium regulation and potentially affect various conditions, including hypertension.

## Effect of the Unfolded Protein Response on Immune Cell Function

The UPR is required for the regulation of the development, differentiation, effector function, metabolism, and survival of adaptive and innate immune cells involved in the pathogenesis of hypertension.

### Adaptive Immunity

#### CD8 + T Cells

**Fig. 4:**
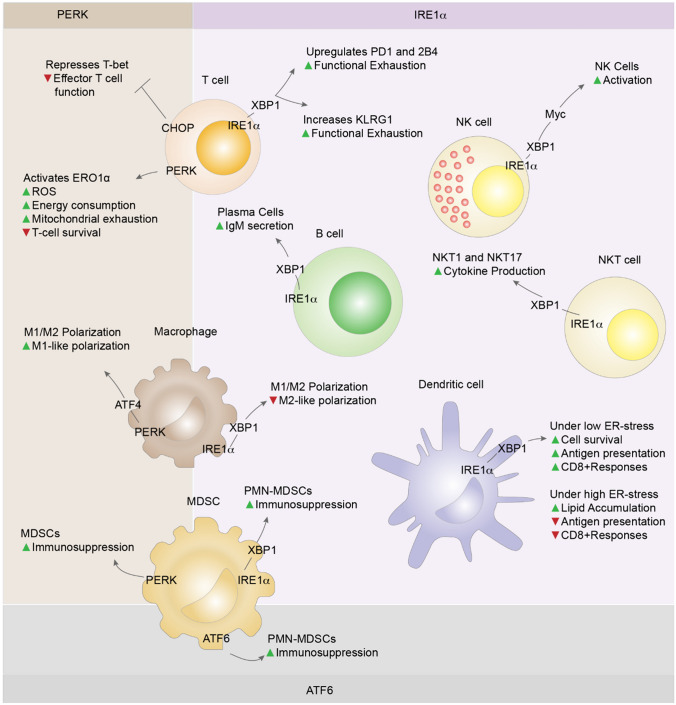
Effects of the UPR on immune cell function. 2B4, Protein; ATF4, Activating Transcription Factor 4; ATF6, Activating Transcription Factor 6; CHOP, C/EBP Homologous Protein; ERO1alpha, Endoplasmic Reticulum Oxidoreductin 1 Alpha; ER, Endoplasmic Reticulum; IgM, Immunoglobulin M; IRE1alpha, Inositol-Requiring Enzyme 1 Alpha; KLRG1, Killer Cell Lectin-Like Receptor G1; M1-like polarization, Macrophage Polarization Type; M1/M2 polarization, Macrophage Polarization Types; MDSCs, Myeloid-Derived Suppressor Cells; Myc, Myelocytomatosis Oncogene; NKT1, Natural Killer T-cell type 1; NKT17, Natural Killer T-cell type 17; PD1, Programmed Cell Death Protein 1; PERK, PKR-like Endoplasmic Reticulum Kinase; PMN-MDSCs, Polymorphonuclear Myeloid-Derived Suppressor Cells; ROS, Reactive Oxygen Species; T-bet, T-box Transcription Factor TBX21; XBP1, X-Box Binding Protein 1

During activation, CD8+ T cells undergo proteome remodeling and increased protein synthesis as they expand and secrete cytokines; this process requires an elevated UPR (Fig. 4**)**. Persistent UPR activation can impair CD8+ T-cell survival and function and lead to functional exhaustion. Hurst et al. reported that PERK activation of ER oxidoreductin 1α (ERO1α), a catalyst for oxidation reduction (redox) reactions, leads to the accumulation of ROS and mitochondrial exhaustion and ultimately impairs survival [93]. Cao et al. reported that CHOP represses the expression of T-bet, a principal regulator of effector T-cell function [94]. In a study of functional exhaustion, Ma et al. demonstrated that cholesterol uptake increases XBP-1 expression in CD8+ T cells, which causes upregulation of inhibitory receptors such as PD-1 and 2B4 and results in a functionally exhausted phenotype. [95]. Kamimura et al. further revealed that XBP-1 and XBP-1 s enhance killer cell lectin-like receptor G1 (KLRG1) expression in CD8 + T cells, predisposing them toward a short-lived, exhausted phenotype [96]. These results are consistent with previous findings that CD8+ T cells with high KLRG1 and low CD127 expression are more prone to functional exhaustion [97•].

This role of the UPR in CD8+ T cells has implications in the context of hypertension, in which T cells play a significant role [98, 99•]. Foundational studies by Guzik et al., Mattson et al., and Crowley et al. revealed that the absence of T and B cells protects against angiotensin-dependent hypertension in RAG1 − / − mice [4], RAG1 − / − Dahl salt-sensitive rats [5], and SCID mouse animal models [6], respectively. However, subsequent studies have reported contrasting findings. Uchida et al. observed no significant difference in Ang II-induced hypertension between wild-type and Rag1 − / − mice [8]. Senchenkova et al. reported that Rag1 − / − mice were not protected from Ang II-induced hypertension but were protected from light-dye-induced thrombosis [9], while IL-6 − / − mice were protected from Ang II-induced hypertension [10]. Mian et al. reported that while Rag1 deficiency did not protect against hypertension, scruffy T-cell transfer reduced blood pressure [11]. Ji et al. noted that Rag1 − / − mice, previously protected from Ang II-induced hypertension, lost protection from Ang II-induced hypertension, suggesting that this could be due to changes in renal AT1R binding due to genetic variation leading to phenotypic changes in Rag1-/- mice since 2015 [12]. Seniuk et al. reported that Rag1 − / − mice were not protected from Ang II-induced hypertension at any dose and that phenotypic changes in Rag1-/- mice may have occurred earlier since 2009 [13•]. In light of these findings, our group previously reviewed these studies to explore these discrepancies [14•], and these findings further underscore the need for additional animal and human studies to confirm a more nuanced understanding of these relationships.

#### CD4+ T Cells

Upon activation, naïve CD4+ T cells differentiate into T helper cells (Th cells; e.g., Th1, Th2, Th9, and Th17 cells), T follicular helper cells, and regulatory T cells (Tregs). Among these cell types, Madhur et al. reported that Th17 cells, which secrete the proinflammatory cytokine IL-17, promote angiotensin-induced hypertension [100]. Barhoumi et al. and others reported that Tregs, which secrete anti-inflammatory cytokines such as IL-10, suppress hypertension [18, 101]. Th17 and Treg differentiation are mutually inhibitory and reciprocally regulated by TGF-β1, affecting the Th17/Treg balance and, consequently, hypertension. Although the role of the Th17/Treg ratio in hypertension has been well established, recent research findings by Bode et al. indicate that deficiencies in complement C3a and C5a receptors implicated in the regulation of Treg conversion do not affect the development of hypertension or hypertensive end-organ damage in Ang II-induced hypertension models. This result underscores the complex interaction between immune system components in hypertension and the need for additional research [15•]. The effect of the UPR on Th17 and Treg differentiation is also not fully understood [102–107]. Therefore, confirmatory experiments involving separate XBP1, PERK, and ATF6 deletions are warranted.

#### B Cells

The UPR plays a critical role in the development and function of B cells, including transitional, naïve, memory, and plasma cells. Elevated protein synthesis is required for plasma cells to secrete antibodies. Nearly 70% of transcripts in long-lived plasma cells are dedicated to immunoglobulin production [108], necessitating enhanced UPR and mTOR metabolic activity. Todd et al. reported that memory B cells develop normally in XBP-1-deficient cells but that plasma cells do not develop normally. These results indicated that XBP-1 is specifically required for immunoglobulin production [109]. Tellier et al. reported that Blimp-1-mediated regulation of XBP-1 is crucial for B-cell antibody secretion and that Blimp-1-deficient cells lose their secretion ability [110].

### Innate Immunity

#### Dendritic Cells

The role of the UPR in DCs is not fully understood; it varies depending on the type of DC, the in vivo or ex vivo setting, the pathological context, and the extent of ER stress [111]. Iwakoshi et al. reported that XBP-1 is critical for DC survival [112]. Osorio et al. reported that XBP-1-deficient DCs have a reduced ability to cross-present antigens to CD8+ cells [113]. Medel et al. reported that activation of the IRE1α-XBP-1 pathway in bone marrow-derived DCs ex vivo is necessary for efficient cross-presentation of melanoma-associated antigens and enhanced CD8+ responses [111]. However, under conditions of high ER stress, such as in the tumor microenvironment, IRE1α has the opposite effect on DCs. Ruiz et al. reported that IRE1α/XBP-1 activation in DCs in the tumor microenvironment promotes aberrant lipid accumulation, diminishes their antigen-presenting capacity, and exerts an immunosuppressive effect. Additional experiments to fully understand the role of the UPR in DCs are warranted [114].

#### Macrophages

The UPR regulates M1/M2 polarization in macrophages. Shan et al. reported that IRE1α affects obesity and metabolic syndrome by affecting macrophage polarization. They found that myeloid-specific ablation of IRE1α leads to an increase in M2 polarization in a cell-autonomous manner and significantly reverses the M1–M2 imbalance induced by a high-fat diet (HFD). These alterations also prevent HFD-induced obesity, insulin resistance, hyperlipidemia, and hepatic steatosis [115]. In a complementary study, Yang et al. reported that PERK induces M1-type polarization in macrophages; this effect was reversed by the PERK inhibitor GSK2656157 [116].

#### Natural Killer Cells and Natural Killer T Cells

Dong et al. revealed that the IRE1α-XBP1-cMyc pathway is crucial for activating mouse and human natural killer (NK) cells. They found that genetic ablation of IRE1α led to a downregulation of cMyc and that overexpression of cMyc could rescue the proliferation defect in IRE1α-deficient NK cells [117]. IRE1α also regulates the function of activated invariant natural killer T cells (iNKTs) by modulating cytokine production. Govindrajan et al. reported that the genetic deletion of IRE1α results in reduced cytokine production in iNKT cells within NKT1 and 17 sublineages [118, 119].

#### Myeloid-Derived Suppressor Cells

The UPR is critical for mediating the immunosuppressive activity of myeloid-derived suppressor cells (MDSCs). Tcyganov et al. reported that the immunosuppressive activity of polymorphonuclear MDSCs (PMN-MDSCs) is mediated by the IRE1α and ATF6 pathways. Inhibition of ER stress reversed the activity of PMN-MDSCs, leading to a tumor-specific immune response and reduced tumor progression. Although PMN-MDSCs depend on the UPR, their study revealed that the activity of monocytic myeloid-derived suppressor cells (M-MDSCs) is independent of the UPR [120•]. Mohamed et al. reported that tumor-infiltrating MDSCs had increased expression of PERK; deletion of PERK reversed these immunosuppressive effects, transforming MDSCs into myeloid cells that activated antitumor CD8+ T-cell responses [121•].

Given the critical role of UPR pathways in the development, differentiation, effector functions, metabolism, and survival of immune cells across both adaptive and innate immunity, UPR modulation has broad implications in the pathogenesis of hypertension.

## Endoplasmic Reticulum Stress and Hypertension

### Endoplasmic Reticulum Stress in Angiotensin II-Induced Hypertension Models

ER stress inhibition attenuates Ang II-induced hypertension. Kassan et al. reported that mice infused with Ang II exhibit a significant hypertensive response compared with control mice, with the systolic blood pressure increasing to 146.9 ± 1.15 mmHg, compared with 100.01 ± 0.58 mmHg in control mice (P < 0.05). The application of ER stress inhibitors, such as taurine-conjugated ursodeoxycholic acid (TUDCA) and 4-phenylbutyric acid (4-PBA), significantly reduced systolic pressure (TUDCA: 108.4 ± 2.1 mmHg; 4-PBA: 111.8 ± 2.16 mmHg). Both inhibitors significantly improve indicators of impaired vascular endothelial function [122].

Young et al. used systemic Ang II administration concurrently with intracerebroventricular infusion of either a control vehicle or TUDCA. Radiometric analysis revealed a pronounced elevation of arterial pressure in the control arm and marked attenuation of hypertension in the TUDCA cohort. [123]. Young et al. further employed a genetic approach in which mice received subfornical organ-targeted microinjections of adenoviral vectors expressing GRP78 to prevent ER stress. Telemetric assessments revealed that the control cohort (injected with a nontherapeutic vector) exhibited prototypical progressive arterial pressure elevation in response to Ang II infusion, whereas the GRP78-overexpressing cohort had no hypertensive response [123].

### Endoplasmic Reticulum Stress in Genetically Hypertensive Models

Chao et al. used male genetically spontaneously hypertensive rats (SHRs) and age-matched Wistar-Kyoto (WKY) rats to evaluate the temporal ER stress profile in the rostral ventrolateral medulla (RVLM) [124]. ER stress in the RVLM preceded the development of hypertension in SHRs. The mRNA and protein levels of GRP78 and p-eIF2α in the RVLM were significantly greater in 6-week-old prehypertensive SHRs than in age-matched WKY rats and remained elevated in 12- and 16-week-old SHRs with established hypertension. Site-specific ER stress reduction was subsequently achieved via 14 days of treatment with salubrinal, an ER stress inhibitor, which resulted in a sustained decrease in the mean arterial pressure in conscious SHRs [124].

Spitler et al. reported that in SHRs, TUDCA and 4-PBA markedly enhanced aortic contractility and reduced oxidative stress compared with those in WKY rats. TUDCA or 4-PBA treatment in SHRs was associated with significant attenuation of cytosolic phospholipase A2 activity, which inhibited the release of arachidonic acid, a crucial component involved in prostanoid synthesis and hypertensive vasoconstriction [125].

### Endoplasmic Reticulum Stress in Deoxycorticosterone Acetate-Salt-Induced Hypertension Models

Jo et al. examined the role of ER stress in deoxycorticosterone acetate (DOCA)-salt-induced hypertension [126]. After three weeks of DOCA-salt treatment combined with intracerebroventricular (ICV) infusion of either artificial cerebrospinal fluid or TUDCA, mice that received ICV infusion of artificial cerebrospinal fluid had significantly increased mean arterial pressure (approximately 14 ± 5 mmHg). ICV administration of TUDCA did not alter this hypertensive response. These findings indicate that mitigating ER stress does not reduce blood pressure in a DOCA-salt-induced hypertension model [126].

Xia et al. reported that mice that received DOCA-salt treatments and were given ICV infusions of TUDCA experienced hypertension progression. These findings indicate that ER stress in the brain does not significantly contribute to the development of DOCA-salt-induced hypertension and suggest that ER stress pathways play different roles in different physiological contexts [127].

### Endoplasmic Reticulum Stress in Dahl Salt-Sensitive Hypertension Models

Yum et al. reported the role of ER stress in the progression of chronic kidney disease in 12-week-old male Dahl salt-sensitive rats. The use of this well-established model of hypertension-associated chronic kidney disease revealed that treatment with 4-PBA effectively reduced hypertension and prevented deterioration of kidney function, as evidenced by reduced proteinuria and albuminuria, both of which are hallmarks of glomerular damage. The protective effects of 4-PBA were superior to those of standard antihypertensive agents (hydralazine and nifedipine). These results emphasize that in addition to its impact on blood pressure, ER stress inhibition has specific renoprotective effects [128].

### Endoplasmic Reticulum Stress in High-Fat Diet Models

Kim et al. reported that, compared with mice fed a regular diet, wild-type mice fed a high-fat diet (HFD) had a notably impaired insulin-induced vasodilation response that was reversed by administration of the ER stress inhibitor TUDCA [129]. Cheang et al. reported that exposure of HFD-induced obese mice to the ER stress inducer tunicamycin resulted in impaired endothelium-dependent relaxation [130], which was reversed by treatment with TUDCA (100 mg/kg per day); additionally, vasorelaxation was markedly improved.

### Endoplasmic Reticulum Stress in Other Hypertension Models

Young et al. reported that acute induction of ER stress via intracerebroventricular administration of the sarcoplasmic/ER Ca2^+^-ATPase inhibitor thapsigargin in adult male C57BL/6 mice leads to significant increases in arterial pressure and renal sympathetic nerve activity [123]. Wang et al. used a Goto-Kakizaki rat model of spontaneous type 2 diabetes mellitus [131]. A subset of the rats underwent aortic coarctation surgery to induce hypertension in the right kidney by creating physical constriction between the left and right renal arteries. After eight weeks, the rats exhibited an average increase in blood pressure from 109 ± 1 mmHg to 152 ± 5 mmHg above the constriction site, which was partially reversed by TUDCA administration (135 ± 4 mmHg vs. 151 ± 4 mmHg); additionally, there were reductions in albumin discharge, ER stress, oxidative stress, and glomerular damage in hypertensive kidneys after treatment [131].

### Endoplasmic Reticulum Stress in Human Studies

Although the relationship between ER stress and endothelial dysfunction has been assessed in human studies, studies on the role of ER stress in hypertension are lacking. Walsh et al. used a randomized, crossover design in which participants in two experimental arms received TUDCA or a placebo [132]. When the participants underwent an oral glucose challenge to induce endothelial dysfunction, those in the TUDCA arm maintained stable endothelial function; control arm participants exhibited prototypical glucose-induced endothelial dysfunction.

Kaplon et al. studied 26 adults who were classified according to body mass index (BMI) as obese (BMI ≥ 30 kg/m^2^, n = 12) or nonobese (BMI < 30 kg/m^2^, n = 14) [133]. Compared with participants in the nonobese group, participants with obesity had greater IRE1, p-PERK, and ATF6 expression and exhibited marked endothelial dysfunction, as evidenced by decreased flow-mediated dilation of the brachial artery. Additional human studies investigating the relationship between ER stress and hypertension are warranted.

## Discussion and Future Directions

This review describes evidence that induction of ER stress leads to a hypertensive phenotype in various animal models, with initial but not confirmatory findings also emerging from limited human studies. Additional research is needed to clarify the mechanisms through which UPR pathways contribute to hypertension, particularly when translating these findings from animal models to human conditions.

While the UPR is a cellular safeguard that regulates proteostasis insults within the ER, exposure to persistent ER stress can cause the UPR to shift to a maladaptive state that exacerbates cellular dysfunction and contributes to the pathology of diseases such as hypertension. Identifying the precise points at which this shift occurs and understanding the triggers that drive the UPR beyond its protective capacity remain crucial areas of research. It is possible that temporal profiling of ER stress and further insights into spatial orientation changes, such as through mass spectrometry imaging techniques, could help identify novel biomarkers for the early detection and intervention needed for hypertension management.

It is crucial to further examine the sequential dynamics of the immune system and ER stress to determine whether these factors predominantly intensify, or initiate, hypertension and associated organ damage and to assess whether many of the immune changes, ER stress, and the UPR are secondary to target organ injury or act as primary drivers. Some studies have shown that protecting organs and blood vessels from elevated blood pressure significantly prevents immune cell infiltration, inflammation, and injury; and that target organ injury, with associated inflammation and immune cell infiltration, can further exacerbate hypertension and target organ damage. The possible presence of a feedback loop requires further study.

Continued examination using advances in imaging techniques to study the structure of the ER and its contact sites across hypertension and salt sensitivity may provide additional mechanistic insights. ER stress can cause the formation of contacts with mitochondria; this coupling can affect cell bioenergetics. As long as proper fixation and quantification considerations are met, electron microscopy can be used to study and quantify these contact sites, along with the use of traditional mitochondrial quantification methods. Three-dimensional techniques can also be used for volumetric analysis of these contact sites. These techniques may offer greater insight into their architecture and involvement in hypertension, if any. Given the link between autophagy and ER stress, identifying and quantifying the autophagic machinery may be equally important topics of study. Thus, understanding how ER stress affects the cellular ultrastructure of both cardiac tissues and immune cells in hypertension may be important for broadening our understanding of the underlying pathomechanisms involved.

Additionally, the interplay between ER stress and other cellular stress responses, such as oxidative stress and inflammation, in hypertension warrants further investigation. These interconnected pathways can provide an improved understanding of the pathophysiology of hypertension and offer new opportunities for therapeutic intervention. ER stress and oxidative stress are often interlinked. The UPR, particularly the PERK-p62-KEAP1-NRF2 pathway, plays a critical role in the modulation of oxidative stress originating from the ER and mitochondria by activating NRF2, the primary regulator of the cellular antioxidant response. We and our collaborators found that an elevated oxidative environment fosters the formation and accumulation of IsoLGs, rapidly creating protein adducts that DCs then present as neoantigens that T cells recognize as nonself. This recognition triggers an immune response and contributes to the progression of hypertension. Given the role of the UPR in managing oxidative stress, modulating the PERK-NRF2 pathway to downregulate IsoLG formation warrants further investigation.

The mechanistic linkage connecting the UPR-immune response-hypertension has also not been fully elucidated. It is possible that such an examination could offer new insights into hypertension pathophysiology and reveal novel therapeutic targets. We previously demonstrated that DC presentation of IsoLG-protein adducts as neoantigens triggers a hypertensive response mediated by T cells. Other groups found that IsoLG-protein adducts initially localize to the ER, triggering ER stress. Whether the UPR or another proteostasis pathway can be modulated to degrade these proteins via an elevated proteasome or ERAD function (before DCs present them to T cells as neoantigens) merits further study.

The role of ER stress in calcium homeostasis is another promising area of investigation. Cellular calcium homeostasis in the cytosol requires a delicate balance between the influx of extracellular calcium into the cytosol and the release of intracellular calcium stores from the ER into the cytosol. ER stress causes a release of calcium sequestered in the ER, leading to an increase in the calcium concentration in the cytosol, partly due to the disrupted function of RyR and IP3R caused by ER stress. Additionally, GRP78, which dissociates from PERK-IRE1α-ATF6 to trigger the UPR, is a major Ca2+ -binding protein that sequesters 25% of the total Ca2+ concentration in the ER. Under ER stress, GRP78 dissociates from Ca2+ in the ER, to which it has a low affinity to act as a molecular chaperone and bind to misfolded and unfolded proteins, for which it has a high affinity, leading to a reduced Ca2+ sequestration capacity in the ER. We found that extracellular calcium influx into the cytosol results in the activation of NADPH oxidase, which produces IsoLGs that ultimately lead to T-cell activation and instigate the formation and activation of the NLRP3 inflammasome, contributing to hypertension. Whether a similar hypertensive effect occurs when ER stress mediates the release of intracellular calcium from ER stores into the cytosol, thus increasing cytosolic Ca2+ concentrations, warrants further investigation.

Given the multifactorial nature of hypertension, it is possible that investigating crosstalk between the UPR and other proteostasis pathways (e.g., heat shock response, autophagy, ubiquitination-related proteasomal activity, the ISR, and the mitochondrial UPR) could offer critical insights. Such an integrative approach could lead to more effective treatments. Given the fundamental role of the UPR in cellular biology, an ideal approach would involve targeting the UPR in a cell-type or organ-specific manner to minimize off-target side effects. Intermittent on/off dosing schedules could also limit toxicity. More research on the interactions of the UPR with proteostasis, oxidative, inflammatory, immune, and metabolic pathways may improve the understanding of the therapeutic potential of targeting ER stress in hypertension and the salt sensitivity of blood pressure.

### Supplementary Information

Below is the link to the electronic supplementary material.

Supplementary file1 (PDF 792 KB)

## Data Availability

No datasets were generated or analysed during the current study.
